# The genetics and epigenetics of satellite centromeres

**DOI:** 10.1101/gr.275351.121

**Published:** 2022-04

**Authors:** Paul B. Talbert, Steven Henikoff

**Affiliations:** Howard Hughes Medical Institute, Fred Hutchinson Cancer Research Center, Seattle, Washington 98109, USA

## Abstract

Centromeres, the chromosomal loci where spindle fibers attach during cell division to segregate chromosomes, are typically found within satellite arrays in plants and animals. Satellite arrays have been difficult to analyze because they comprise megabases of tandem head-to-tail highly repeated DNA sequences. Much evidence suggests that centromeres are epigenetically defined by the location of nucleosomes containing the centromere-specific histone H3 variant cenH3, independently of the DNA sequences where they are located; however, the reason that cenH3 nucleosomes are generally found on rapidly evolving satellite arrays has remained unclear. Recently, long-read sequencing technology has clarified the structures of satellite arrays and sparked rethinking of how they evolve, and new experiments and analyses have helped bring both understanding and further speculation about the role these highly repeated sequences play in centromere identification.

Centromeres are the genomic loci where the proteinaceous kinetochores are assembled to attach to spindle microtubules to orchestrate chromosome segregation during mitosis and meiosis. In most organisms, a single centromere is found on each chromosome at a specific location. The location of centromeres is widely viewed as an epigenetic phenomenon independent of DNA sequence (Karpen and Allshire 1997) and instead dependent on the location of nucleosomes containing the centromere-specific variant of histone H3 (cenH3), known as CENPA or CENP-A in animals (Earnshaw and Rothfield 1985) or CENH3 in plants (Zhong et al. 2002), which specifies kinetochore assembly. Yet centromeres in animals and plants are usually located within species-specific, very highly repeated tandem head-to-tail arrays of noncoding sequences known as satellites, which typically comprise both the centromere and the flanking pericentromere of animal and plant chromosomes (Plohl et al. 2014). Satellite arrays have been called the “dark matter” (Ahmad et al. 2020) of the genome because of the difficulty of assembling blocks of sequences that are identical or nearly so, leaving large gaps in chromosome assemblies. In recent years, however, long and ultralong sequencing reads from Pacific Biosciences (PacBio) SMRT technology and Oxford Nanopore Technologies have cast illumination on previously dark matter, allowing assembly of previously intractable arrays. Whereas short sequencing reads have defined the point centromeres of budding yeast (Fitzgerald-Hayes et al. 1982) and short regional centromeres of unicellular eukaryotes (Sanyal et al. 2004; Kanesaki et al. 2015), long reads have helped to assemble the transposon-rich centromeres of fungi (Sonnenberg et al. 2020) and satellite centromeres in maize (Wolfgruber et al. 2016; Liu et al. 2020). The use of long-read sequencing technologies recently allowed the completion of the telomere-to-telomere (T2T) assembly of an entire human genome (Nurk et al. 2022), more than 17 years after the human genome project was declared to be complete, and following quickly on the completion of the human X Chromosome (Miga et al. 2020) and Chromosome 8 (Logsdon et al. 2021), the first two human chromosomes to be completely sequenced. Although analysis of human centromeres of chromosomes other than 8, X, and Y (Jain et al. 2018) has not been completed, these T2T assemblies will allow us to see how satellite families and subfamilies are arranged and offer insight into their evolution and functions.

The very first sequenced centromeres (Fitzgerald-Hayes et al. 1982) from budding yeast are occupied by a single CENPA-like nucleosome (Furuyama and Biggins 2007; Henikoff and Henikoff 2012) and are generally regarded as genetic centromeres, because they contain binding sites for specific DNA-binding proteins that can self-assemble the kinetochore, including the kinetochore-specifying cenH3. However, the view that other centromeres are predominantly epigenetic is supported by much evidence, including the occurrence of human neocentromeres, in which CENPA nucleosomes located on previously noncentromeric, nonsatellite sequence can support kinetochore function, and by the occurrence of pseudo-dicentric chromosomes, in which there is one active and one suppressed centromere (Karpen and Allshire 1997). In addition, centromeric sequences are known to evolve rapidly and differ dramatically between sibling species (Henikoff et al. 2001), suggesting that DNA sequence conservation does not exist for this essential function in every cell cycle. More recently, insect holocentromeres, centromeres that occupy large chromosomal regions instead of a specific locus, have been found to lack CENPA (Drinnenberg et al. 2014), depending instead on other kinetochore proteins including the histone fold domain protein CENPT (Cortes-Silva et al. 2020). *Bombyx* holocentromeres occupy large domains of inactive chromatin covering half of the genome (Senaratne et al. 2021). These centromere domains can be lost or gained in response to nearby gene activation or silencing. These observations and others have been interpreted to mean that DNA sequence does not matter for most centromeres. Yet this leaves unexplained why the vast majority of natural animal and plant centromeres occupy large satellite arrays, and why satellite centromeres seem to be restricted to animals and plants and are not found in fungi or other eukaryotes (Talbert and Henikoff 2020).

## Why satellite arrays?

**Figure 1.**
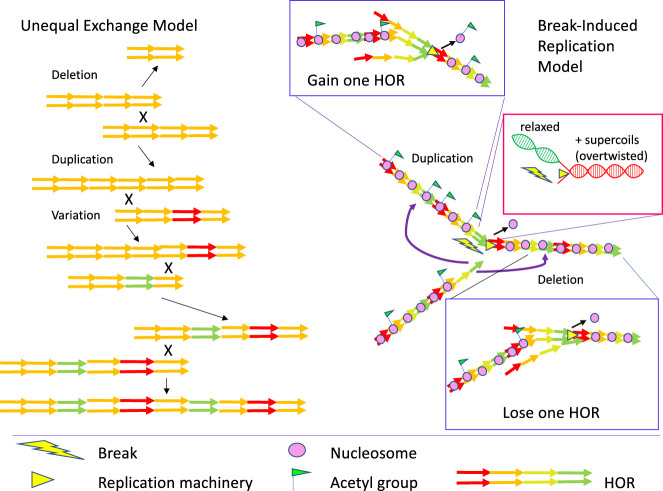
Models of amplification of higher-order repeats (HORs). (*Left*) In the unequal exchange model, reciprocal recombination between out-of-register tandem repeats can either duplicate or delete individual monomers. As variations accumulate in particular monomers, unequal exchange can generate higher-order repeats (HORs). (*Right*) In the break-induced replication (BIR) model, replication fork stalling can lead to one-ended double-strand breaks (DSBs). Resection yields a free single-strand 3′ end that can invade a homologous sequence and reinitiate replication. Reinitiating at an out-of-register repeated sequence ahead of the fork will lead to deletion, whereas reinitiating at one behind the fork will lead to duplication (*insets* with blue outlines). Duplication appears to be favored, perhaps because the chromatin behind the fork is more accessible to strand invasion owing to the new acetylated histones and/or the relaxed torsional state in contrast to the overtwisted DNA ahead of the fork (*inset* with red outline).

A potential explanation for the existence of satellite arrays was proposed in the unequal exchange model (Smith 1976). In this model (Fig. 1), once a tandem duplication is established through periodicities generated by random mutation followed by unequal exchange between sister chromatids that does not require extensive homology, the resulting duplication can undergo unequal out-of-register exchange with its copy on the sister chromatid (or homolog), generating further reciprocal duplications and deletions. As mutation alters the sequence of individual monomers, they can become encompassed within higher-order repeats (HORs), in which sets of distinct monomers are duplicated together to form larger repeats. With an exchange rate high enough, significant homogeneity can be maintained in the face of mutation. This model is neutral, in that there is no preference for preserving duplications rather than deletions, and if an array is deleted down to one monomer the process is extinguished, suggesting a need for some mutational or selective force to maintain or expand the array to generate the natural arrays of megabases of repeats.

Dover (1982) emphasized the importance of gene conversion, a form of nonreciprocal recombination between homologous sequences in which a donor sequence replaces or “converts” another sequence, in homogenizing families of repetitive sequences, particularly when they are physically close, as in tandem arrays. Dover viewed gene conversion, unequal exchange, and transposition as processes that turn over DNA and can be stochastic or directional, which he termed molecular drive. He proposed that the accumulation of such changes and homogenization within populations could lead to “accidental speciation” due to incompatibility between separate populations, offering a rationale for the long-held suspicion that satellite arrays have a role in speciation (Yunis and Yasmineh 1971; Ferree and Prasad 2012).

The discovery of the single-stranded annealing pathway, which repairs double-strand breaks (DSBs) by annealing distinct homologous repeat sequences and deleting sequences between them (Lin et al. 1984), suggested that tandem satellite arrays would shrink over time unless a process favoring expansion counteracted this repair pathway. A Darwinian process that favors expansion of centromeric arrays was proposed in the centromere drive model (Henikoff et al. 2001). In this model, the observed rapid evolution of both centromeres and kinetochore proteins such as CENPA and CENPC (another foundational kinetochore protein) was proposed to result from a genetic conflict between satellite DNA variants acting selfishly to favor their own transmission through female meiosis and kinetochore proteins evolving to suppress this biased transmission. Because female animals and plants have asymmetric meiosis in which only one of the four meiotic products is transmitted to the next generation, centromere variants will compete for inclusion in the egg or megaspore, and variants that can attract more kinetochore proteins will have “stronger” centromeres that can favor their orientation at the first meiotic division so that they end up in the egg rather than in a polar body. Such lack of parity between centromeres may cause problems from unequal tension in male meiosis, in which all four meiotic products contribute to fertility, and so it is hypothesized that kinetochore proteins evolve to suppress centromere drive by restoring parity between homologs. The ensuing rapid divergence of centromeres and kinetochore proteins was proposed as a possible mode of generating incompatibilities that result in speciation (Henikoff et al. 2001). In contrast to a purely epigenetic view of centromere specification, this model implies that variant satellite arrays differ genetically in their ability to recruit kinetochore proteins. Centromere drive therefore can be viewed as favoring genetic control by a satellite variant over the assembly of kinetochore proteins, especially cenH3, whereas suppression can be viewed as a disruption of variant-specific interactions to make kinetochore assembly insensitive to driving genetic variants and restore a more epigenetic or DNA-sequence-independent mode of kinetochore assembly (Dawe and Henikoff 2006). Strong supporting evidence for centromere drive has been found in monkeyflowers (*Mimulus sp*.), in which the large satellite duplication *D* can be transmitted to 90% of offspring through female meiosis but male meiosis follows Mendel's rules (Fishman and Willis 2005; Finseth et al. 2015), and in which there has been a recent selective sweep of a cenH3 allele that modifies drive (Finseth et al. 2021). Additionally, mice chromosomes with more centromeric repeats load more CENPA and are preferentially segregated to the egg (Chmátal et al. 2014; Iwata-Otsubo et al. 2017). Similarly, Robertsonian fusions in humans, which combine two acrocentric centromeres into a stronger metacentric centromere, are preferentially transmitted in females but not in males (Pardo-Manuel de Villena and Sapienza 2001).

The centromere drive model predicts that variant centromeres that acquire more kinetochore proteins will be favored in female meiosis in plants and animals, but it does not tell us what features of satellites in particular are favored. Although satellite monomers come in a range of lengths, sizes approximating the length of one or two nucleosomes predominate (Melters et al. 2013). Satellites impose translational and rotational phasing on nucleosomes (Hasson et al. 2013; Zhang et al. 2013; Henikoff et al. 2015), generating regular nucleosome arrays and potentially a regular kinetochore structure. Many satellites have a 10-bp periodicity of WW (W = A or T) dinucleotides (Talbert and Henikoff 2020), which favors rotational phasing and minimizes the bending energy of wrapping DNA around nucleosomes (Prytkova et al. 2011; Struhl and Segal 2013), making nucleosomes more stable. This greater stability may be important to form a strong kinetochore under the tension exerted by microtubules during anaphase I, so that selection would favor the expansion of structurally suitable sequences.

## Insights from long sequencing reads

Can the fully assembled centromere sequences available from long-read sequencing technologies tell us more about how satellites evolve or why they are favored in evolution? One of the first satellite centromeres to be assembled to near completion using PacBio long reads was the 1.85 Mb *centromere chromosome 10* (*cent10*, also known as *CEN10*) from maize (Wolfgruber et al. 2016). This study uncovered evidence of frequent recombination events mediated by microhomology, including a hemicentric inversion that split the original array of *centc* (the maize centromeric satellite, also known as *CentC*), internal deletions in centromeric retrotransposons (CRs), recombination between nearby retroelements, insertion of mitochondrial sequences, and adjacent duplications. The investigators argued that these events are better explained by microhomology-mediated end-joining, a mode of error-prone DSB repair, than by unequal exchange or gene conversion.

More recently, long-read sequencing has allowed assembly of seven maize centromeres, including T2T assemblies of Chromosomes 3 and 9 (Liu et al. 2020). Three centromeres lack *centc* entirely, being composed of the CR transposons that target centromeres and other transposons. In these maize centromeres there does not appear to be any preference by CENH3 for *centc* versus other sequences. This lack of correlation may be explained because inbreeding and selection for centromere-linked genes during domestication greatly reduced *centc* and the number of surviving haplotypes while simultaneously selecting for the fixation of at least 57 distinct neocentromeres (Schneider et al. 2016).

Human centromeres are made up of α-satellite, with monomers of ∼171 bp. Most monomers fall into two types, A and B (Alexandrov et al. 2001). Type A monomers have a 19-bp motif called an n box (Rice 2020b) that overlaps the binding site for a protein of unknown function pJα (Gaff et al. 1994), and B monomers have in the corresponding location a 17-bp binding site called the CENP-B box or simply the B box, which is bound by CENPB, the only known sequence-specific human kinetochore protein (Masumoto et al. 1989). A and B monomers usually occur in alternation (Alexandrov et al. 2001). A and B monomers are arranged in HORs such that whereas individual pairs of monomers within a HOR may be only 50%–70% identical, copies of a particular multimeric HOR are usually nearly identical. The edges of satellite arrays have disordered monomeric satellites (Schueler et al. 2001; Miga et al. 2020; Logsdon et al. 2021), whereas the middle of arrays consist of HORs, of which the simplest is a dimer of A and B monomers. In an analysis of PacBio reads and consistent with earlier results, CENP-B boxes were most frequently found in every other monomer, that is, as part of n/B dimers (A/B dimers), and only rarely were found in adjacent monomers (Rice 2020b). The reason that CENP-B boxes seem to be disfavored in adjacent monomers is not clear, but they may be constrained by the additional kinetochore proteins that form complexes on the n/B dimers (Thakur and Henikoff 2018) and the unknown role of the pJα protein that binds n boxes (Gaff et al. 1994).

The n/B dimers can be subdivided into families by their degree of sequence similarity, with Suprachromosomal Family 1 (SF1) dimers found on Chromosomes 1, 3, 5, 6, 7, 10, 12, 16, and 19, and Suprachromosomal Family 2 (SF2) dimers on Chromosomes 2, 4, 8, 9, 13, 14, 15, 18, 20, 21, and 22 (Alexandrov et al. 2001; Henikoff et al. 2015). The SF3 family consists of pentamers found on Chromosomes 11, 17, and X, and as an additional HOR on Chromosome 1. Usually only one HOR on each chromosome can form the centromere (McNulty and Sullivan 2018). The simple SF1 or SF2 n/B dimer structure forms the basis of the longer chromosome-specific HORs, which may have 2, 3, or 4 n/B dimers, or in longer HORs the dimer structure may be interrupted by additional monomers. In some dimers, especially in longer HORs, a CENP-B box may be mutated so that it no longer binds CENPB, and there may be noncanonical monomers. A notable exception to the n/B dimeric structure of centromeric satellites is the Y Chromosome, which has A monomers with n boxes but which lacks B monomers and CENP-B boxes, and has the longest human HOR at 34 monomers (Jain et al. 2018). Although unequal exchange or gene conversion may contribute to homogenizing HORs in satellite arrays, the complex structures of HORs have been more difficult to explain by these models.

## The break-induced replication (BIR) model

In analyzing long reads from human HORs enriched in CENPA, Rice (2020b,c) concluded that the complex nested structures of HORs could be created by break-induced replication (BIR). In this model, the constitutive centromere associated network (CCAN), the persistent core of the kinetochore present throughout the cell cycle in most animals, presents a barrier to replication as it does in yeasts (Greenfeder and Newlon 1992; Mitra et al. 2014), which results in fork pausing and collapse, creating a one-ended DSB (Fig. 1). Indeed, human α-satellites are enriched for aphidocolin-sensitive DSBs, indicative of replication stalling (Crosetto et al. 2013). Restarting collapsed forks is carried out by the BIR pathway (Sakofsky and Malkova 2017). Resection of the one-ended break allows the free 3′ strand of the truncated sister chromatid to reinitiate replication on its sister chromatid by BIR or microhomology-mediated BIR (Kockler et al. 2021). Initiation will frequently be out-of-register in a tandem array, with initiation behind the fork leading to expansion of the array, and initiation ahead of the fork leading to deletion (Fig. 1). In yeast rDNA arrays, expansion by BIR is favored over deletion (Kobayashi 2014), suggesting a preference for initiation behind the fork. Newly replicated chromatin is hyperacetylated and depleted for histone H1 relative to bulk chromatin (Perry and Annunziato 1989), whereas parental chromatin in front of replication forks is positively supercoiled (overtwisted). The relatively increased accessibility behind the replication fork would facilitate strand invasion and favor repeat expansion.

In the BIR model (Rice 2020c), HORs are hypothesized to go through a “life cycle” starting with n/B dimers, which are favored by centromere drive because binding of CENPB enhances recruitment of CENPC, making a stronger centromere and increasing the fidelity of centromere function (Fachinetti et al. 2015). Centromere drive acting on CENP-B boxes has also been proposed as the explanation for why the Y Chromosome lacks CENP-B boxes, because it never experiences centromere drive in female meiosis (Marshall and Choo 2012). From an n/B dimer, additional dimers can be added to make longer HORs, which are favored because their greater length allows them to expand laterally more quickly and to occupy the central core of the satellite array more easily than an equivalent number of dimers, pushing out older HORs to the sides of the kinetochore, where they decay over time because they are no longer subject to frequent BIR (Rice 2020c). However as HORs increase in length they are also more likely to acquire CENP-B box mutations, additional n-box monomers, or other divergences that make them susceptible to replacement by a young n/B dimer HOR, perhaps inserted from a different chromosome by template switching. Besides potentially accounting for the expansion of highly identical HORs, BIR is also mutagenic, with elevated levels of frameshifts and base substitutions that are 500-fold or more greater than in normal S-phase replication (Sakofsky and Malkova 2017). Error-prone BIR therefore may account for the rapid divergence of centromeric HORs at the nucleotide level, which is estimated to be greater than 10 times the divergence on chromosome arms between humans and chimps (Rice 2020a). This is consistent with the view that elevated mutation rates at the point centromeres or short regional centromeres of yeasts (Padmanabhan et al. 2008; Bensasson 2011) may be the result of fork stalling (Greenfeder and Newlon 1992; Mitra et al. 2014) followed by BIR repair.

## Do T2T assemblies of human centromeres support the BIR model?

**Figure 2.**
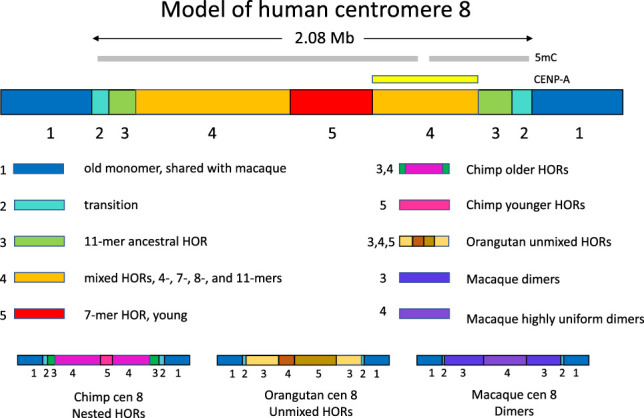
Human centromere 8. (*Top*) Human centromere 8 shows successive evolutionary layers (1–5), with the oldest monomer layer on the edges of the array and the youngest, most uniform HOR in the middle (Logsdon et al. 2021). (*Bottom*) Similar structures are seen in great apes, whereas rhesus macaque centromere 8 consists entirely of dimers most closely related to the old monomers.

Many features of the BIR model are supported by the recently completed T2T assembly of human Chromosome 8 (Logsdon et al. 2021). The investigators of this study compared the 2-Mb centromeric alpha satellite array with Centromere 8 assemblies from chimpanzees, orangutans, and rhesus macaques and found that each of these primate centromeres showed a largely symmetrical satellite array with four or five layers of evolutionary structure, with each layer similar on the p and q arms (Fig. 2). The α-satellite monomers in the flanking pericentromeres of humans and chimps (layer 1) fall into two clades, one of which is present only in the q arm and which has common ancestors with monomers and dimers from macaque, indicating an ancient stratum of the α array. The second human layer is a short (∼60 kb) transitional region between monomers and HORs. The third human layer is composed largely of an 11 monomer HOR. The large fourth layer has the greatest variety of HOR subtypes including HORs of 4, 7, and 8 monomers intermixed with the 11 monomer HOR from which they are derived. The fifth layer is a 177-kb region entirely composed of nearly identical 7 monomer HORs. The HORs of great apes all have a common origin distinct from α monomers, and chimpanzees and gorillas resemble humans in having similar transitional layers from monomers to HORs with different arrangements of blocks of HORs in subsequent layers, whereas macaques have a large central block of highly uniform dimers flanked by more divergent dimers. An elevated mutational divergence was found between centromeres, two- to fourfold higher than at random loci, consistent with an error-prone repair process such as BIR. The investigators proposed a model in which highly identical repeats expand, pushing older repeats out of the centromere. They hypothesized that the more divergent clade of monomers shared between macaques and the q arm of apes represents the remnants of the ancestral centromere.

In apparent conflict with the predictions of the BIR model, the 632-kb region of CENPA on Chromosome 8 was found not in the fifth layer of nearly identical 7-mers but in the adjacent fourth layer in a “high entropy” region of great admixture of HOR types, which the investigators suggest may reflect “potential optimization of HOR subtypes associated with the active kinetochore” (Logsdon et al. 2021), although it is unclear why this admixture would be optimal. This result appears to be in contrast to ChIP mapping of CENPA to the 12-mer DXZ1 HOR on the X and to recently homogenized dimers on other chromosomes, where CENPA occupancy falls off exponentially with divergence from the consensus (Henikoff et al. 2015). On the Y Chromosome, CENPA occupancy is largely coincident with the 34-mer DYZ3 HOR array, although reduced CENPA occupancy was also found in flanking divergent alpha satellite up to 20 kb on either side of the HOR (Jain et al. 2018). Both the DXZ1 and DYZ3 arrays encompass some interspersed minor HOR variants, indicating that homogenization within arrays can be imperfect (Jain et al. 2018; Miga et al. 2020). It may be relevant that in non-satellite centromeres of horse and donkey, epialleles of a 100-kb CENPA block were found in various locations within a few hundred kilobases in different individuals and were mitotically inherited but could “slide” in position over one generation (Nergadze et al. 2018). These investigators suggested that satellite arrays may reduce the impact of such positional flexibility.

An intriguing feature of the DNA methylation patterns in active human centromeres is a hypomethylated region, known as the centromeric dip region, within a hypermethylated HOR that is occupied by CENPA (Gershman et al. 2022). The hypomethylated regions have higher nucleosome density, reduced methylation of CENP-B boxes, low accessibility, and correspond to peaks of CENPA occupancy, suggesting that these regions may be inaccessible to methyltransferases because they are protected by the kinetochore. Hypomethylation of satellite repeats occupied by CENH3 compared with the same repeats in flanking heterochromatin was also reported in *Arabidopsis*, maize, and *cen11* of rice, although other rice centromeres that have more transposons and less of the satellite *CentO* showed elevated methylation instead (Zhang et al. 2008; Yan et al. 2010). In contrast, the HORs of human X and 8 centromeres are essentially devoid of transposons yet are mostly methylated except in the centromeric dip region.

## Replication of α-satellite

**Figure 3.**
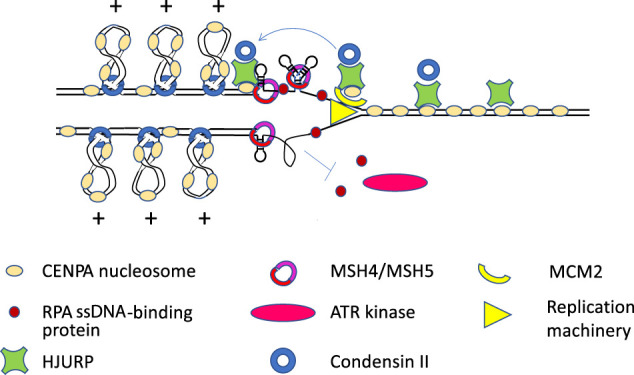
Speculative model of replication through α-satellite. Holliday junction recognition protein (HJURP) associates with CENPA nucleosomes before S-phase and recruits the condensin II complex. At the replication fork, HJURP and the MCM2 subunit of the replication machinery work together to assure that CENPA nucleosomes reassemble behind the fork. DNA secondary structures form on single-stranded repetitive DNA behind the fork, and HJURP and mismatch repair proteins (MSH4 and MSH5 are shown) bind to them and resolve them. Condensin II complexes extrude positively supercoiled DNA loops, and the positive torsion inhibits the binding of replication protein A (RPA), which binds single-stranded DNA and must accumulate in order for the ATR serine/threonine kinase (ATR) to signal that DNA damage has occurred and to arrest replication. This inhibition by condensin II allows time for secondary structures to be resolved. Condensin II is also needed with HJURP to assemble new CENPA nucleosomes in G1, and condensin-mediated loops may play a role in the organization of the kinetochore.

Although the BIR model is supported by the high mutation rate in centromeres and the structure of HORs and their evolutionary layers in human centromere 8, an apparent conflict exists with the assumption of the model that the CCAN causes replication stalling and breakage in satellite centromeres. Contrary to expectation, depletion of CENPA greatly increases fork stalling in human centromeres with increased unequal exchange and formation of R-loops, likely caused by replication-transcription conflicts, followed by unfinished replication and anaphase bridges or by breakage and translocations at centromeres (Giunta and Funabiki 2017; Giunta et al. 2021). This does not preclude a role for the CCAN in causing fork stalling and BIR, but it indicates that satellite centromeres face additional more serious causes of fork stalling in repeated sequences when CENPA and the CCAN are reduced. Mismatch repair proteins that bind to four-stranded Holliday junctions and their single-stranded progenitor structures such as DNA hairpins (Snowden et al. 2004) are enriched in replicating α-satellite that has been introduced into *Xenopus* egg extracts, suggesting that DNA secondary structures form in single-stranded repetitive DNA behind the replication fork (Aze et al. 2016), with the potential to contribute to fork stalling if they interfere with DNA polymerization. Positively supercoiled DNA and chromatin loops are also enriched in replicating α-satellite, dependent on topoisomerase I, which acts together with condensins to introduce positive supercoils into DNA (Hirano 2012). Positive supercoiling suppresses the accumulation of the single-strand binding protein, replication protein A (RPA), which can activate ATR serine/threonine kinase (ATR)-dependent DNA-damage-checkpoint signaling. This supercoiling-dependent suppression gives time for secondary structures to be resolved, facilitating replication through α-satellite (Aze et al. 2016). Centromeres are enriched during interphase in the condensin II complex, which is necessary for proper CENPA loading and retention (Bernad et al. 2011) and is mutually interdependent for centromeric localization with Holliday junction recognition protein (HJURP), the chaperone that assembles CENPA into centromeres during G1 (Barnhart-Dailey et al. 2017) and which is necessary to retain CENPA through replication (Zasadzińska et al. 2018). HJURP has been suggested to interact with the mismatch repair protein MSH5 (Kato et al. 2007) and can bind to DNA (Müller et al. 2014) and possibly to structured DNA such as Holliday junctions in vitro (Kato et al. 2007), suggesting a possible role for the secondary structures that form on replicating α-satellite in directing or supporting HJURP's role in retaining CENPA through replication (Fig. 3).

## What does CENPB do?

The BIR model proposes that n/B dimers were acquired through centromere drive and are the foundation from which other HORs are built, presumably because CENPB strengthens the kinetochore. What exactly does CENPB do? CENPB is a protein that is a domesticated transposase that has lost transposase activity (Smit and Riggs 1996; Kipling and Warburton 1997). It is conserved throughout mammals, but CENP-B boxes are present in the centromeric satellite arrays of only some mammalian clades, such as great apes, mice, and horses, but not in old world monkeys, rabbits, carnivores, and others (Gamba and Fachinetti 2020). The function of CENPB in clades that lack CENP-B boxes is unknown, whereas the nine bases required for CENPB binding appear to have evolved independently in each lineage that has CENP-B boxes, leading to the observation that CENPB appears to have evolved to stabilize kinetochore function in preexisting satellite centromeres (Gamba and Fachinetti 2020).

In vitro CENPB binds the CENP-B box in the major grooves and is able to kink the DNA with a bend of 59° (Tanaka et al. 2001). It forms antiparallel homodimers that can bind two CENP-B boxes at once and can form loops between CENP-B boxes on the same DNA molecule (Yoda et al. 1998). In cells, the acidic domain of CENPB has seemingly conflicting functions promoting both kinetochore formation and heterochromatin formation through different interacting partners (Otake et al. 2020). CENPB binds to both the CENPA amino-terminal tail and to CENPC and is necessary to maintain proper levels of CENPC (Fachinetti et al. 2015). Neocentromeres and the Y Chromosome centromere, both of which lack CENP-B boxes, have reduced levels of CENPC and have increased levels of chromosome mis-segregation compared to other centromeres, consistent with the view that CENPB makes stronger centromeres that are favored by centromere drive.

In the prevailing epigenetic templating model of CENPA localization and maintenance, CENPA recruits CENPC, which recruits the M18BP1 licensing complex and the CENPA chaperone HJURP to load new CENPA next to its preexisting locations in a self-dependent loop (for review, see McKinley and Cheeseman 2016). Using an auxin-inducible degron system that destroys existing CENPA, Hoffmann et al. (2020) found that new CENPA localized back to the same HORs in native centromeres. This localization depended on the ability of DNA-bound CENPB to bind to CENPC, on the recruitment of the M18BP1 licensing complex and HJURP by CENPC and on the loading of new CENPA by HJURP. Thus, de novo CENPA deposition did not depend on preexisting CENPA at centromeres. Using a lacO system to tether CENPB to an ectopic site, the investigators showed that CENPB could recruit CENPC and CENPA, but CENPA recruitment was dependent on CENPC and could not be recruited directly by CENPB. Although nearly 100% of cells recruited new CENPA to native centromeres in the presence of CENPB, in the absence of CENPB ∼40% of centromeres were still able to partially load de novo CENPA, and de novo CENPA was loaded onto ∼25% of Y Chromosomes, suggesting that α-satellite has some ability to recruit CENPA even without CENPB, but that preexisting CENPA probably also contributes to maintaining CENPA at the Y centromere via the M18BP1 licensing complex. These results are consistent with the long-held observation that human artificial chromosomes with functioning centromeres can be made from α-satellite HORs that contain CENPB boxes (Ohzeki et al. 2002, 2020). These observations indicate that human centromeres have a genetic component in the same sense as budding yeast centromeres in that CENPB is able to bind the centromere and assemble a kinetochore, analogous to the sequence-specific DNA-binding proteins of yeast.

## Do other sequences besides the CENP-B box matter?

Prominent phasing of CENPA nucleosomes occurs on HORs of both the X and Y Chromosomes, although phasing is more precise on the X, suggesting that CENP-B boxes are unnecessary for phasing but contribute to its precision (Hasson et al. 2013), probably by direct contact between bound CENPB and the amino terminus of CENPA. By mapping CENPA ChIP-seq reads onto PacBio reads, long arrays of Centromere 1-like dimers (SF1) and Centromere 13-like dimers (SF2) were found to comprise most active centromeres and to precisely position CENPA and CENPC on each monomer in the dimer, with a CENP-B box between them (Henikoff et al. 2015). CENPA and CENPC occupancy of dimers was reduced by as little as 2%–10% divergence from the consensus sequences of SF1 and SF2 dimers. In a follow-up study, high salt extraction released intact particles containing CENPA/B/C that probably represent the intact CCAN (Thakur and Henikoff 2018). Enrichment of these particles correlated with the density of CENP-B boxes in different HORs, although lower enrichment of CENPA-containing particles was also found on sequences with few or no CENP-B boxes, such as the D7Z2 HOR of Chromosome 7. Mapping of fragments onto SF1 dimer arrays revealed a 50-fold difference in occupancy of different dimers and a diversity of structures. For example, mapping to four adjacent dimers of D7Z1 that are 88%–96% identical, particles were found on both monomers or only one monomer of a dimer. In the latter case, the CCAN particles could overlap the CENP-B box either from the left or right. These observations suggest that very similar sequences can dramatically affect occupancy by the CCAN, which appears to be flexible in conformation.

## Non-B form DNA in centromeric satellites

HORs and CENP-B boxes characterize satellite arrays in great apes, but in other organisms both satellite and nonsatellite centromeres are enriched in dyad symmetries that are predicted to form non-B form DNA structures such as cruciforms or hairpins (Koch 2000; Kasinathan and Henikoff 2018). Short (<10 bp) dyad symmetries that are predicted to extrude cruciform structures are common features in the α-satellite of old world monkeys, in the human Y centromere, in human and chicken neocentromeres, and in the centromeres of horses, chickens, plants, and fission yeast (Kasinathan and Henikoff 2018). In contrast, the CENP-B-box-containing α-satellite of great apes and mouse centromeric satellite are predicted to have a low propensity to form cruciforms, but genome-wide mapping using permanganate treatment in the human and mouse genomes (Kouzine et al. 2013, 2017) nevertheless revealed non-B form DNA in these centromeres that correlated with CENPA enrichment (Kasinathan and Henikoff 2018). CENPB can bend DNA by 59° (Tanaka et al. 2001), and this may enhance cruciform formation by CENP-B-box-containing repeats. Such secondary structures may be a defining feature directing CENPA deposition. The CENPA chaperone HJURP was originally identified as a protein that interacted with mismatch repair proteins and could bind four-way DNA junctions in vitro (Kato et al. 2007), and it is possible that it recognizes cruciform structures in centromeres and/or the mismatch repair proteins that bind them and deposit CENPA. It is unknown whether its distant fungal homolog Scm3 also binds four-way junctions, but Scm3 homologs in various fungi contain AT hooks, myb domains, and zinc fingers (Aravind et al. 2007) that might impose or stabilize cruciform structures on transposons or other centromeric sequences. In this way, either spontaneous or induced cruciforms would constitute sequence-encoded features targeted by CENPA chaperones. These structural features could be the raw material on which centromere drive acts.

## Perspective

Long-read sequencing has made it possible to know the complete structures of satellite centromeres, and although only a few are known so far, the structures have brought into question the long accepted but seldom carefully examined unequal exchange model for their evolution. Evidence for microhomology-based repair mechanisms has been invoked from both maize and human centromeres, and further evaluations of repair and recombination mechanisms in satellites are warranted as well as better understanding of the elevated mutational rates in centromeres of all types. With tools such as degron and tethering systems, the genetic properties of human centromeres and the role of CENP-B boxes have been clarified, and these and other tools promise further progress in understanding the interactions between centromeres, kinetochores, chaperones, replication, and transcription in mitosis and meiosis.

The development of tools to better predict and map non-B DNA structures and supercoiling in centromeres could possibly change the way we think about centromere specification. The ability to form non-B DNA from a variety of sequences, including both native centromeres and sequences that become neocentromeres, could unite the genetic and epigenetic views of centromeres. Non-B DNA provides a large sequence space from which centromeric DNA can be selected and may provide a rationale for why centromeres are usually formed on AT-rich DNA (Talbert and Henikoff 2020), which melts more easily and could aid in forming transient cruciforms or other secondary structures. Such structures might contribute to the fork stalling, breakage, error-prone repair, expansions, and rearrangements that occur at centromeres, the processes that make centromeres the most evolutionarily dynamic structures in the genome.
